# Factors influencing rational antibiotic use behaviours of early childhood caregivers in three tertiary care hospitals, Bangkok metropolitan: a cross-sectional descriptive study

**DOI:** 10.1080/20523211.2025.2581101

**Published:** 2025-11-04

**Authors:** Supreeda Manipantee, Jintana Kasemsiri

**Affiliations:** Department of Pediatric Nursing, Kuakarun Faculty of Nursing, Navamindradhiraj University, Bangkok, Thailand

**Keywords:** Health literacy, Antibiotic use behaviour, Rational antibiotic use, Caregivers of early childhood

## Abstract

**Background:**

The increasing prevalence of antimicrobial resistance (AMR) is a global health issue, and one of the key contributing factors is the inappropriate and excessive use of antibiotics. This problem is particularly relevant to early childhood, where antibiotic administration relies heavily on caregivers. Despite its critical importance, research on the factors influencing rational antibiotic use behaviours among early childhood caregivers remains limited in Thailand. This study, therefore, aims to explore caregivers’ behaviours in administering antibiotics to children, as well as personal and health literacy factors that may influence these behaviours.

**Method:**

This cross-sectional descriptive study was conducted in 2024 among 183 caregivers of children under 6 years of age who visited the pediatric outpatient clinics at three tertiary hospitals in Bangkok Metropolitan. Rational antibiotic use was measured using a questionnaire with a 5-point Likert scale. Descriptive statistics (including percentage, mean, and standard deviation) and multiple linear regression analyses were used to explore the relationship between caregivers’ characteristics, antibiotic literacy, and rational antibiotic use behaviour.

**Results:**

The overall rational antibiotic use behaviour among caregivers was at a moderate level (mean 3.08, S.D.  0.56). However, inappropriate behaviours were also observed, with 18.6% of caregivers reporting immediate antibiotic administration for fever and 12.6% saving leftover antibiotics. Multiple linear regression analysis revealed that caregivers’ household income, knowledge, self-management, and decision-making abilities collectively predicted rational antibiotic use behaviour, accounting for 13.4% of the variance (*p* < .001).

**Conclusions:**

This study concludes that caregivers’ rational antibiotic use needs to be improved. Household income, knowledge, and self-management skills, including decision-making, are key factors influencing this behaviour. The findings have practical implications and can be applied to develop educational programmes and policies that enhance caregivers’ antibiotic literacy and promote safe, rational antibiotic use.

## Background

Antimicrobial resistance (AMR) poses a global health challenge that affects everyone. Focusing on developing future therapeutic and preventive approaches is essential to mitigate its widespread effects (Das et al., 2024). In Thailand, AMR trends are increasing, with nearly 100,000 cases of antimicrobial resistance infections and approximately 38,000 associated deaths annually. This results in an economic loss of at least 40 billion THB, equivalent to nearly 1% of the national Gross Domestic Product (GDP). The primary cause of AMR is the inappropriate and excessive use of antibiotics (Sumpradit et al., 2021). Early childhood is particularly vulnerable due to underdeveloped immune systems, making them more susceptible to infections. It has been found that antibiotics were used in children, with 43.7% administered for respiratory tract infections and 35% for gastrointestinal infections (Sridhamma et al., 2021).

Additionally, research has shown that there are misconceptions about antibiotic use among caregivers, with 77% believing that antibiotics can eliminate both bacteria and viruses, and 32.5% reporting that they would stop administering antibiotics once symptoms improved (Kwannate & Tachasuksri, 2019). Under these circumstances, the Ministry of Public Health has adopted measures aligned with the World Health Organization's guidelines on the rational use of medicines and has launched public education campaigns. A national goal has been set to achieve 30% antibiotic literacy among the population by 2027. However, current data show that only 14% of the population demonstrates adequate literacy in rational antibiotic use (Thammathatcharee et al., 2022).

A literature review on inappropriate antibiotic use behaviour has revealed several patterns, including the overuse of antibiotics or use without medical indication, failure to complete prescribed courses, and self-medication (Yana & Chaisombut, 2021). Factors that influence such behaviour include a caregiver’s age and educational level, which are positively associated with health literacy in taking care of early childhood (under 6 years of age). It was found that caregivers aged 31–40 years with at least a secondary education demonstrate an ability to seek, evaluate, and apply health information appropriately in childcare decisions (Faengphong et al., 2023). Rational Drug Use (RDU) literacy refers to an individual’s ability to access health information and services, comprehend relevant knowledge, communicate effectively, manage self-care, make informed decisions, and critically assess media content in order to appropriately administer antibiotics to children (Nunkong, 2023). Income is also another related factor, as a study on the relation of rational drug use within a population showed that individuals with higher income tend to have better access to quality healthcare services, increasing the likelihood of receiving appropriate prescriptions (Saengungsumalee et al., 2024). However, some research indicated that household income is not significantly associated with caregivers’ antibiotic use behaviours for school-aged children (Kwannate & Tachasuksri, 2019).

While most existing studies have focused on antibiotic use among the general population, particularly adolescents and adults, limited research specifically examines caregivers’ behaviour regarding antibiotic use in early childhood and its influencing factors, especially in the context of Thailand. This is a crucial knowledge gap, as the administration of antibiotics to young children is largely at the discretion of their caregivers. The average number of drugs per prescription in Bangkok is approximately 2.17 items, which is higher than the WHO-recommended range of 1.6–1.8 items (Khangtragool et al., 2023). Therefore, this study aims to investigate the antibiotic use behaviour of early childhood caregivers, with a particular focus on identifying key influencing factors, including age, educational level, household income, and rational antibiotic use literacy. The findings are expected to contribute to the development of targeted strategies to promote rational antibiotic use among caregivers, thereby supporting better health outcomes and quality of life for children.

## Method

### Study design

This cross-sectional descriptive study collected data using questionnaires from caregivers of children aged from birth to under 6 years. The study was conducted at the pediatric outpatient departments of three tertiary hospitals in Bangkok Metropolitan.

### Setting

The study was conducted at three tertiary care hospitals in Bangkok Metropolitan. These three hospitals were purposively selected from a total of ten public hospitals under the Bangkok Metropolitan Administration, representing all tertiary care hospitals in this jurisdiction that provide specialised pediatric outpatient services

### Study population and sample size

The study population consisted of caregivers of children from birth to under 6 years of age. A total of 183 caregivers were recruited using a convenience sampling method, with 61 participants from each hospital. The sample size was determined using G*Power 3.1 software (Faul et al., 2009) based on power analysis, with a medium effect size (*f*² = 0.15), a significance level (Alpha) of 0.05, and a power of 0.80 (Cohen, 1992). For nine predictor variables, the minimum required sample size was 166. An additional 10% was added to account for potential incomplete or missing data, resulting in a final total sample size of 183 participants.

### Inclusion and exclusion criteria

The inclusion criteria for caregivers were defined as parents, grandparents, or relatives who were the primary individuals responsible for the child's daily care and health. They must have brought their child to the pediatric outpatient departments at the study sites and be able to understand and communicate effectively in Thai. The exclusion criteria were children with severe or chronic illnesses that required urgent medical attention or those whose clinical symptoms worsened during the study period.

### Data collection procedures

Upon receiving institutional and ethical approval, the researcher obtained permission from the heads of the pediatric outpatient departments to collect data. The researcher provided participants with a detailed explanation of the study objectives and procedures and obtained their informed consent before data collection began. The questionnaire was self-administered for literate participants and took approximately 15–20 min to complete. For participants who were unable to read, the researcher read the questions aloud and recorded their responses. Data collection was carried out between May and September 2024. All data was collected using paper-based questionnaires and treated confidentially. Participants received monetary compensation of 300 THB for their time.

### Research instrument and validation

The research instrument consisted of three main parts. Part 1, General Information, included questions on the caregiver's age, education level, and household income. Part 2, Health Literacy, was a validated questionnaire from the Health Education Division, Department of Health Service Support, Ministry of Public Health et al. (2018). It assessed six aspects: access to health information, cognitive, communication skills, self-management, decision-making skills, and media literacy. Part 3, Rational Antibiotic Use Behaviour, was adapted from the assessment tool by Sornkrasetrin et al. (2019) and included both positive and negative questions.

All questions were rated using a 5-point Likert scale. For health literacy, scores ranged from 5 (very high) to 1 (very low). For rational antibiotic use behaviour, scores ranged from 5 (always) to 1 (never). The instrument's quality was verified by assessing content validity with five experts, yielding an Item-Content Validity Index (I-CVI) of 0.83. The questionnaire was then pre-tested with 30 caregivers of early childhood, yielding a Cronbach's Alpha Coefficient Reliability of 0.89. The questionnaire is included in the supplementary files.

### Data analysis

The data collected was analysed using SPSS for Windows. Descriptive statistics, including frequencies, percentages, means, and standard deviations, were used to summarise the characteristics of the participants, health literacy, and rational antibiotic use behaviour. An overall health literacy score and a rational antibiotic use behaviour score were computed by averaging the scores from their respective sub-components.

To analyse factors influencing rational antibiotic use, a multiple linear regression analysis was performed. Rational antibiotic use behaviour was the dependent variable, while the caregivers’ characteristics and their health literacy aspects were the independent variables. Variables with a *p*-value of less than 0.05 were included in the final regression model.

## Results

### Participant characteristics

A total of 183 individuals were contacted to participate in the study, and all of them consented and were included in the research. There were no refusals or withdrawals. The mean age of the caregivers was 35.77 years (S.D. = 11.39), with 95% CI of 34.12–37.42. The majority of the participants were female (89.1%), while 10.9% were male. Most participants (54.6%) were between 30 and 44 years of age. Regarding education, the highest proportion of caregivers (27.3%) had completed upper secondary school/vocational certificates or held a bachelor's degree. The average monthly household income was 29,331.42 THB (S.D. = 23,712.4), with 95% CI of 25,896.36–32,766.48. A significant proportion of participants (50.3%) had never brought their child for healthcare services at the hospital before. The detailed sociodemographic data are presented in Table 1.

**Table 1.** Sociodemographic data among caregivers of early childhood (*n* = 183).

Information	Number (persons)	Percentage (%)
**Age of caregivers**		
19–29 years	53	29
30–44 years	100	54.6
45–59 years	19	10.4
>60 years	11	6
$\bar{X}$ = 35.77 S.D. = 11.39 Min = 19 Max = 79 95% CI (34.12–37.42)		
**Sex**		
Female	163	89.1
Male	20	10.9
**Experience bringing a child to a hospital for healthcare services**		
Never used the service	92	50.3
Have used the service before	91	49.7
**Educational level**		
Never attended school	4	2.2
Primary school	15	8.2
Lower secondary school	37	20.2
Upper secondary school/vocational certificate	50	27.3
Diploma or equivalent/higher vocational certificate	22	12
Bachelor’s degree or equivalent	50	27.3
Master’s degree or higher	5	2.7
Household income (THB/Month)*		
< 10,000	16	8.7
10,001–20,000	67	36.6
20,001–30,000	50	27.3
> 30,001	50	27.3
$\bar{X}$ 29,331.42 S.D. = 23,712.4 Min = 0 Max = 200,000 95% CI (25,896.36–32,766.48)		

*The exchange rate for US Dollar (USD) to Thai Baht (THB) during the study period (May–September 2024) was approximately 36.50–37.00 THB per 1 USD.

### Health literacy

**Figure 1.**
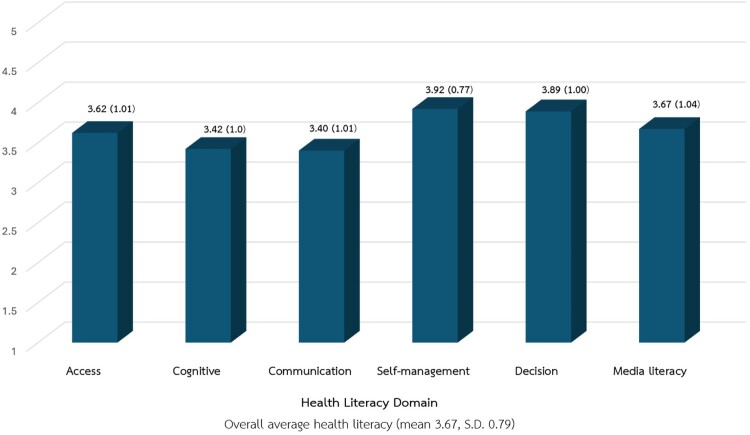
Mean scores and standard deviations of six health literacy domains (*n* = 183).

The overall health literacy of the caregivers was at a high level (mean 3.67, S.D. 0.79). As shown in Figure 1, among the six components, self-management received the highest mean score (mean 3.92, S.D.0.77), indicating a strong ability among caregivers to manage their health needs. The next highest score was in decision-making (mean 3.89, S.D. 1.00), suggesting that caregivers are proficient at making informed health-related choices. In contrast, the communication aspect scored the lowest (mean 3.40, S.D.1.01), which may point to a need for improvement in their knowledge and skills to communicate effectively about appropriate antibiotic use with others.

### Rational antibiotic use behaviours

Overall, the caregivers’ behaviour regarding the rational use of antibiotics was found to be at a moderate level (mean 3.08, S.D. 0.56). Behaviours that majority of caregivers demonstrated appropriately include reading medication labels and instructions before administration (83.1%), strictly administering antibiotics to children following doctors’ prescriptions (81.4%), giving antibiotics on schedule (80.9%), checking expiration dates before use (78.7%), and discontinuing antibiotics and consulting a doctor immediately when signs of allergy appear (77%). Furthermore, most caregivers reported never crushing antibiotic tablets or applying the powder to wounds to fasten healing (82.5%) and did not adjust the dosage or switch antibiotics on their own when the child's condition worsened or did not improve (80.3%). However, some inappropriate practices were also observed. For example, 18.6% of caregivers administered antibiotics as soon as the child developed a fever accompanied by clear nasal discharge to accelerate healing. 12.6% reported saving leftover antibiotics for further use if the child presented with similar symptoms, and 6% gave antibiotics when the child experienced nausea and vomiting due to food poisoning. These findings can be found in Table 2.

**Table 2.** Mean scores, S.D., frequencies and percentages of rational antibiotic use behaviours among caregivers of early childhood (*n* = 183).

Rational Antibiotic Use Behaviours	Always (%)	Quite often (%)	Often (%)	Some times (%)	Never (%)	$\bar{X\;}$	S.D
**Overall rational antibiotic use behaviour**						**3.08**	**0.56**
Strictly administer antibiotics to children following doctors’ prescriptions	81.4	14.8	3.3	0.5	0	4.77	0.53
Read medication labels and instructions before administration	83.1	13.7	3.3	0	0	4.80	0.48
Check the expiration date of the antibiotic every time before giving it to children	78.7	10.9	7.1	1.6	1.6	4.63	0.82
Discontinue antibiotics and consult a doctor immediately when signs of allergy appear (e.g. rash, hives)	77	15.8	2.7	2.7	1.6	4.64	0.81
Request antibiotics from the doctor even when informed that they are not necessary	8.7	7.7	4.9	10.9	67.8	1.79	1.33
Stop antibiotics once symptoms improve, even before completing the full course	6.6	13.7	5.5	17.5	56.8	1.96	1.33
When the doctor prescribes antibiotics and the child’s condition does not improve after 1–2 days, change the doctor or buy a stronger drug for the child	8.2	7.7	3.3	15.3	65.6	1.78	1.30
Increase dosage of antibiotic by themselves when illness becomes more severe	4.4	6.0	3.8	5.5	80.3	1.49	1.10
Change the type of antibiotic without medical advice if current medication seems ineffective	5.5	7.1	2.7	4.4	80.3	1.53	1.18
Administer antibiotics on schedule as prescribed by doctor	80.9	15.8	0.5	0.5	2.2	4.73	0.71
Continue administering antibiotics for the full prescribed duration, even if symptoms improve	71.0	16.9	4.9	6.0	1.1	4.51	0.92
Check the physical appearance of the antibiotics to ensure they are not degraded before usage	53.6	29.0	11.5	3.8	2.2	4.28	0.96
Store antibiotics appropriately (e.g. away from light, heat, humidity; refrigerate reconstituted suspensions)	68.3	23.5	3.8	1.1	3.3	4.52	0.88
Save leftover antibiotics for further use in case of similar illnesses	12.6	13.1	6.0	16.4	51.9	2.18	1.49
Administer antibiotics immediately as soon as the child developed a fever accompanied by clear nasal discharge	18.6	15.8	8.7	15.3	41.5	2.55	1.59
Once noticing a sore throat or red throat, let the child drink water and get enough rest before seeing a doctor or pharmacist to consider antibiotic use	34.4	31.7	13.1	9.8	10.9	3.69	1.33
Give oral rehydration solution and soft foods to children without antibiotics when having loose stools not exceeding 3 times a day, with no mucus and no fever	19.7	25.7	16.4	16.4	21.9	3.05	1.45
Give antibiotics to children when having nausea and vomiting from food poisoning	6.0	6.6	13.7	12.6	61.2	1.84	1.24
Give antibiotics to children every time there is a bleeding wound to prevent infection	3.3	4.4	7.1	14.8	70.5	1.55	1.03
Crush antibiotic tablets or applying the powder on the wounds	1.6	3.3	4.4	8.2	82.5	1.33	0.84

### Predictive factors of rational antibiotic use behaviour

Stepwise multiple regression analysis revealed that the independent variables could explain 13.4% of the variance in rational antibiotic use behaviour among caregivers of early childhood (*F* = 8.026, *p* < .001). Significant predictors included household income (*β* = .252, *p* < .001), health literacy in the aspects of cognitive (*β* = .261, *p* = .003), self-management (*β* = .197, *p* = .038), and decision-making (*β* = .171, *p* = .040), as shown in Table 3.

**Table 3.** Predictive factors of rational antibiotic use behaviour among caregivers of early childhood in three tertiary hospitals, Bangkok (*n* = 183).

Variable	B	SE_b_	β	t	*P*-value
Household income	5.597E-6	.000	.252	3.583	<.001**
Cognitive	.138	.046	.261	3.013	.003**
Self-management	.134	.064	.197	2.088	.038*
Decision-making skills	.091	.044	.171	2.071	.040*
Constant = 3.706, R square = .153, Adjust R square = .134, F = 8.026 (df 4, 178), *p* < .001					

*Statistically significant at the .05 level. **Statistically significant at the .01 level.

## Discussion

This study aimed to investigate the level of rational antibiotic use among caregivers of early childhood and to identify the predictive factors influencing their behaviour. The key findings of this study were that while caregivers demonstrated a moderate level of rational antibiotic use, certain behaviours remained inappropriate. Additionally, household income and specific health literacy components were identified as significant predictors of this behaviour.

### Rational antibiotic use behaviours

The study found that caregivers generally demonstrated appropriate antibiotic use behaviours, such as reading medication labels, administering the correct dosage, and adhering to the prescribed schedule. However, a proportion of caregivers still administered antibiotics immediately upon the beginning of illness, due to the belief that antibiotics can eliminate all types of infections. This often led to unnecessary use of antibiotics and the practice of saving leftover antibiotics for further use. These findings align with those of Kwannate and Tachasuksri (2019), which reported that appropriate behaviours of parents for school-aged children included taking the child to see a doctor in the event of an allergic reaction to antibiotics (72.5%), immediately discontinuing the medication when allergic symptoms appeared (68.5%), and ensuring the child completed the full course of antibiotics as instructed by healthcare professionals (67%). In contrast, inappropriate practices observed in their study included administering antibiotics for symptoms such as cough and sore throat (43%) and diarrhea (41%). In this study, caregivers were family members, which may have contributed to a higher level of attention to the child’s health. In Thai culture, there is a high level of trust in healthcare professionals. As a result, caregivers tend to strictly follow medical advice. This differs from studies in Italy which reported that parents administered antibiotics to children without a physician’s prescription (10.4%) (Pierantoni et al., 2021). Similarly, in Kosovo, parents were found to request antibiotics from physicians or obtain them without a prescription (28.9%), and some even recommended the same antibiotics to friends whose children exhibited similar symptoms (Imeri et al., 2023). These inappropriate behaviours highlight a persistent misunderstanding of when antibiotics are truly necessary.

### Factors influencing rational antibiotic use behaviour

The study identified several factors that significantly predicted rational antibiotic use behaviour among caregivers. The overall model was statistically significant, *F*(4, 178) = 8.026, *p* < .001, and accounted for 13.4% of the variance in antibiotic use behaviour (Adjusted *R*^2^ = .134). The analysis revealed that household income and several aspects of health literacy, including cognitive, self-management, and decision-making skills, were significant positive predictors of rational antibiotic use behaviour. This indicates that caregivers with higher household income and better skills in these areas tended to exhibit more appropriate antibiotic use practices.

**Household income:** Household income was found to be a significant positive predictor (*β* = .252, *p* < .001). This suggests that caregivers with higher incomes are more likely to exhibit appropriate antibiotic use behaviours. This is consistent with a study by Saengungsumalee et al. (2024), which also found that higher income was associated with better access to medication and rational use. Higher-income caregivers tend to seek immediate medical attention for even minor illnesses, ensuring they receive proper treatment and guidance on antibiotic use. Conversely, caregivers with lower incomes are more likely to self-medicate or use leftover antibiotics for minor illnesses to avoid the cost and time commitment of a hospital visit, which could interrupt their work. This behavioural pattern increases the risk of inappropriate antibiotic use. While individuals in Thailand, including those with lower incomes, can access government facilities, the findings of this study suggest that socioeconomic factors and the need to maintain employment significantly influence health-seeking behaviours and, consequently, the appropriateness of antibiotic use.

**Health literacy:** The health literacy components cognitive skill (*β* = .261, *p* = .003), self-management skill (*β* = .197, *p* = .038), and decision skill (*β* = .171, *p* = .040) were all identified as significant positive predictors of rational antibiotic use behaviour. This means that caregivers with higher levels of these specific health literacy skills were more likely to use antibiotics appropriately.

#### Cognitive skill and public health efforts

Caregivers with a strong cognitive skill (*β* = .261, *p* = .003) were more likely to administer antibiotics correctly by demonstrating an understanding of essential information, such as how to mix medications, give them orally, and store them properly. This finding is highly consistent with studies linking antibiotic knowledge to correct use behaviour (Özdemir et al., 2023; Yana & Chaisombut, 2021). The level of cognitive skill observed in this population is likely supported by two key factors: successful exposure to national public health campaigns, like the government’s Antibiotic Smart Use (ASU) programme, and the caregiver’s educational status. Analysis of the data confirmed that educational status was significantly and positively associated with cognitive skills, indicating that higher formal education strongly supports the acquisition of crucial health knowledge needed for rational antibiotic use. Despite these support factors, the fact that cognitive skill remains a significant predictor in the regression model suggests that current campaigns, while effective in providing fundamental knowledge, still need enhanced strategies to ensure that this knowledge is translated into consistent, practical behaviour. Future efforts should focus on closing the gap between knowing the facts and applying them correctly in complex, real-life caregiving situations.

#### Self-management

Self-management skill was identified as a significant positive predictor of rational antibiotic use behaviour (*β* = .197, *p* = .038). This skill is critical as it reflects the caregiver’s capacity to plan, schedule, and adhere to the complex antibiotic regimen, ensuring correct timing and dosage throughout the full course. This emphasis on practical adherence aligns with findings that effective caregiver self-management directly supports proper child healthcare practices (Potipiti, 2022). Furthermore, the literature strongly reinforces that a high degree of parental self-efficacy, a core component of self-management, reduces the risk of inappropriate practices. Conversely, poor self-management, such as storing leftover antibiotics or obtaining them without a prescription, significantly increases the likelihood of misuse (Cruz et al., 2022). These results underscore that empowering caregivers with concrete skills to manage medication logistics and control access is a key determinant for achieving rational antibiotic use.

#### Decision-making skills

Similarly, decision-making skills were a significant positive predictor (*β* = .171, *p* = .040). This skill reflects the caregiver's ability to critically evaluate information and engage in informed discussions with health professionals, considering both benefits and risks before making treatment choices. This capacity for making sound choices is crucial because high decision-making skills help caregivers manage the uncertainty and anxiety surrounding their child's illness, thereby reducing the impulse for inappropriate actions like requesting unnecessary antibiotics or self-medication (Marsh et al., 2024). These results affirm that empowering caregivers with critical thinking and effective communication is essential for rational antibiotic use.

#### Negative findings and non-significant predictors

This study also revealed that several personal factors, such as the caregiver's age and educational level, did not significantly influence rational antibiotic use behaviour. This may be attributed to the context of the study population, which comprised caregivers accessing services at a tertiary care hospital in an urban area. In such settings, participants have greater access to immediate and accurate health information directly from medical professionals, which may mitigate the influence of pre-existing personal factors on their behaviour. This finding aligns with the study by Porisutiwutiporn and Hemchayat (2014), which similarly reported that age and education did not significantly influence appropriate antibiotic use among the general population.

Furthermore, other components of health literacy, including access to health information and services, communication skills, and media literacy, were also not found to have a significant influence on caregivers’ behaviour. The non-significance of these factors is likely due to the high level of access and resources available to this urban population. Caregivers in this setting were able to receive health information and direct communication from healthcare professionals effortlessly. They were also exposed to information via various advanced channels provided by the hospital, such as digital media and telemedicine, which diminishes the impact of a lack of media literacy or access barriers. This finding contrasts with studies conducted in rural areas, such as the one by Nunkong (2023), where media literacy was found to have a statistically significant positive relationship with rational antibiotic use, highlighting the difference in environmental and resource factors between urban and rural settings.

## Conclusions

The overall findings of this study indicate that caregivers of early childhood in Bangkok exhibit a moderate level of rational antibiotic use behaviour, highlighting a critical gap between knowledge acquisition and consistent practical application in a resource-rich, urban setting.

The multivariate analysis confirms that household income, cognitive, self-management, and decision-making skills together significantly predict rational antibiotic use, accounting for approximately 15.3% of the behavioural variance (*R*^2^ = .153, *F*(4,178) = 8.026, *p* < .001). Individually, all three Health Literacy Skills emerged as significant positive predictors. These results strongly suggest that public health efforts must transcend basic factual knowledge and emphasise skill-based empowerment. Specifically, promoting self-management skill is vital for adherence and controlling medication access, while fostering decision skill is necessary to manage anxiety and ensure effective communication with healthcare providers. The positive link between educational status and cognitive skill further underscores the need for tailored interventions. These findings affirm the foundational role of national initiatives like the Antibiotic Smart Use (ASU) programme, while simultaneously offering crucial policy direction: to enhance ASU's effectiveness, public communication must shift its emphasis from general knowledge to specific, actionable skills. This insight serves as a foundation for both public and private institutions to develop targeted behaviour change programmes that lead to more responsible antibiotic use.

### Strengths and limitations

The primary methodological strength of this study lies in its focused analysis of the three core Health Literacy skills (cognitive, self-management, decision-making), providing a nuanced understanding of caregiver behaviour that is highly valuable for designing actionable, skill-based interventions, thus enhancing the validity of our findings. However, the cross-sectional design prevents establishing cause-and-effects. Crucially, reliance on self-reported behaviour carries the risk of social desirability bias, which may have overestimated the reported prevalence of rational antibiotic use. Additionally, the convenience sample from a single urban tertiary hospital limits the generalizability of the findings, particularly to rural or low-resource settings.

### Recommendations

Routine Practice: Targeted skill-based training at care points: healthcare providers should design specific interventions at discharge or follow-up visits to develop caregivers’ self-management. These should focus on practical activities such as using visual medication schedules and establishing clear protocols for storage and disposal of leftover antibiotics. Enhance communication and anxiety management: clinical communication training for physicians and nurses should emphasise supporting caregivers’ decision-making skills by proactively addressing parental anxiety about the child's illness and managing their expectations to reduce the inappropriate demand for unnecessary antibiotic prescriptions. Ensure information readability and personalisation: due to the strong link between educational status and cognitive skills, all patient education materials should utilise plain language principles and be routinely tested for readability to ensure accessibility, particularly for parents with lower formal education.

Future Research: Interventional Studies: Future research should employ Randomised Controlled Trial (RCT) designs to rigorously test the effectiveness of the skill-based interventions (focusing on self-management and cognitive skills) on improving rational antibiotic use behaviour, thereby establishing cause-and-effect relationships. Comparative Contextual Studies: Conduct research utilising the same Health Literacy framework in rural or low-resource communities to compare the impact of Health Literacy skills across populations with varying levels of resource access, addressing the limitations of generalizability identified in this study.

## Data Availability

The analysed data are available in the form of questionnaire documents and can be accessed upon request.
